# Tumor budding cells, cancer stem cells and epithelial-mesenchymal transition-type cells in pancreatic cancer

**DOI:** 10.3389/fonc.2012.00209

**Published:** 2013-01-04

**Authors:** Eva Karamitopoulou

**Affiliations:** Clinical Pathology Division, Institute of Pathology, University of BernBern, Switzerland

**Keywords:** pancreatic cancer, epithelial–mesenchymal transition, cancer stem cells, tumor budding

## Abstract

Pancreatic ductal adenocarcinoma (PDAC) is one of the most lethal cancers with a 5-year survival rate of less than 5%. Moreover, PDAC escapes early detection and resists treatment. Multiple combinations of genetic alterations are known to occur in PDAC including mutational activation of KRAS, inactivation of p16/CDKN2A and SMAD4 (DPC4) and dysregulation of PTEN/PI3K/AKT signaling. Through their interaction with Wingless-INT pathway, the downstream molecules of these pathways have been implicated in the promotion of epithelial–mesenchymal transition (EMT). Emerging evidence has demonstrated that cancer stem cells (CSCs), small populations of which have been identified in PDAC, and EMT-type cells play critical roles in drug resistance, invasion, and metastasis in pancreatic cancer. EMT may be histologically represented by the presence of tumor budding which is described as the occurrence of single tumor cells or small clusters (<5) of dedifferentiated cells at the invasive front of gastrointestinal (including colorectal, oesophageal, gastric, and ampullary) carcinomas and is linked to poor prognosis. Tumor budding has recently been shown to occur frequently in PDAC and to be associated with adverse clinicopathological features and decreased disease-free and overall survival. The aim of this review is to present a short overview on the morphological and molecular aspects that underline the relationship between tumor budding cells, CSCs, and EMT-type cells in PDAC.

## GENERAL OVERVIEW

Pancreatic ductal adenocarcinoma (PDAC) is a common cause of cancer death and has a very poor prognosis (Hidalgo, 2010). Most patients present with advanced stage disease and have a median survival of less than 1 year (Stathis and Moore, 2010). Surgical resection is the only potentially curative treatment of PDAC. Classical histomorphological features like tumor size, blood vessel or lymphatic invasion and presence of lymph node metastases constitute essential prognostic determinants in pancreatic cancer and are invariably included in the pathology reports, with tumor stage being the most important of all (Fernandez-del-Castillo et al., 2012). The lethal nature of PDAC has been attributed to the propensity of PDAC cells to rapidly disseminate to the lymphatic system and distant organs (Li et al., 2012).

However, even patients with completely resected, node-negative PDACs eventually die of their disease. Within this context and considering the fact that the management of PDAC remains suboptimal and that adjuvant therapy has resulted to limited progress, the identification of additional reliable and reproducible prognostic markers that would enable better patient stratification and eventually provide a guide towards a more successful and individualized therapy, is mandatory (Welsch et al., 2007; Hidalgo, 2010).

## MOLECULAR CHARACTERISTICS OF PANCREATIC CANCER

Multiple combinations of genetic alterations are known to occur in PDAC including mutational activation of oncogenes like KRAS (>90% of PDACs), inactivation of tumor suppressor genes like TP53 (75–85%), p16/CDKN2A (40%), and SMAD4 (DPC4; 60%) and dysregulation of PTEN/PI3K/AKT signaling (Hruban et al., 2012). Especially the transforming growth factor-beta (TGF-β) is an important and commonly deregulated signaling pathway in pancreatic carcinomas. Alteration of this pathway has a prominent function in both the tumor cell and stromal cell compartments (McCleary et al., 2012). SMAD4 inactivation is present in more than half of all pancreatic tumors, and this phenomenon appears to be specific for pancreatic adenocarcinoma (Schutte et al., 1996; Wilentz et al., 2000). Through a functional TGF-β signaling pathway and the presence of activating KRAS mutations, TGF-β ligand stimulation promotes tumor cells to undergo epithelial–mesenchymal transition (EMT) and thus develop an aggressive and invasive phenotype (Ellenrieder et al., 2001).

## EPITHELIAL–MESENCHYMAL TRANSITION (EMT)

Epithelial–mesenchymal transition is a biologic process essential for embryonic processes like gastrulation and reflects a reversible embryonic program, which is considered a critical feature of normal development and allows partial or complete transition between an epithelial and a mesenchymal phenotype. If aberrantly activated acts as a trigger of tumor progression and metastasis (Kalluri, 2009; Thiery et al., 2009). During EMT epithelial cells undergo morphologic changes characterized by a transition from an epithelial to a mesenchymal phenotype, leading to increased migratory capacity and invasiveness (Karamitopoulou et al., 2011). The hallmark of EMT is the down-regulation of the cell adhesion molecule E-cadherin, which is a transmembrane protein essential for the stable adherens junctions, and the up-regulation of the mesenchymal molecules vimentin, fibronectin, and/or N-cadherin. It has been reported that repression of E-cadherin is associated with dedifferentiation, infiltrative growth, and high incidence of lymph node metastasis in pancreatic cancer, as well as various other malignancies (Hase et al., 1993; Tanaka et al., 2003; Schmalhofer et al., 2009). In more details, EMT is activated by key signaling pathways, including the TGF-β pathway, converging in the stimulation of EMT activators, a group of transcription factors repressing epithelial gene expression. These include members of the snail family, of the bHLH family and of the zinc finger homeodomain (ZFH) family (ZEB1 and ZEB2; Thiery et al., 2009). In many cancers, including pancreatic cancer, EMT has been shown to correlate with high-grade malignancy including the competence to form metastasis (Krantz et al., 2012).

Epithelial–mesenchymal transition occurs at the invasive front of cancers and is essentially reversible by a process called mesenchymal-to-epithelial transition (MET; Thiery and Sleeman, 2006; Wu and Zhou, 2009). In pancreatic cancer, it was shown to be an independent indicator of poor prognosis (Masugi et al., 2010).

Epithelial–mesenchymal transition activators not only activate cellular motility, but are also associated with the maintenance of stem cell properties and cell survival (Mani et al., 2008; Morel et al., 2008). Moreover, EMT has been linked to cellular self-renewal programs of cancer stem cells (CSCs) and apoptosis resistance, which are also features of therapy resistance (Krantz et al., 2012).

## CANCER STEM CELLS

Cancer stem cells are defined by their immortality, their capacity to reproduce all derived cell phenotypes of a cancer and by biological and biochemical markers such as CD44, CD133, aldehyde, dehydrogenase, etc. Stem cell populations have been identified in PDAC representing less than 1% of the total (Floor et al., 2011). These cells show a triple positive phenotype for CD44/CD24/EpCAM and are 100-fold more tumorigenic than the other neoplastic cells (Floor et al., 2011). Recently, *in vitro* studies have suggested that CSCs and EMT-type phenotypes overlap and that the properties of CSCs and EMT-type cells may be linked through shared molecular features (Floor et al., 2011).

## TUMOR BUDDING IN PDAC

Tumor budding corresponds to a type of diffusely infiltrative growth observed in many gastrointestinal cancers (including oesophageal, gastric, colorectal, and ampullary cancers) and is defined as the presence of detached isolated single cells or small cell clusters (up to five cells) scattered in the stroma at the invasive tumor margin (Prall, 2007). The identification of tumor budding is highly facilitated by immunostaining with cytokeratin which helps to better recognize and visualize the buds. Aim of the tumor buds seems to be the degradation of the peritumoral connective tissue, the evasion of host’s response and finally the invasion of the lymphatic and blood vessels with the consequence of local and distant metastasis (Lugli et al., 2012a). To achieve this aim tumor buds have to detach themselves from the main tumor body by loss of membranous expression of the adhesion molecule E-cadherin. Indeed, aggressive, dissociated tumor buds not only lose membranous E-cadherin, but also express fibronectin within the cytoplasm implying a more mesenchymal phenotype underlining the interaction between tumor buds and the surrounding stroma (Kirchner and Brabletz, 2000). Moreover, tumor budding cells have been shown to express nuclear β -catenin which implicates the Wingless-INT (WNT) signaling pathway in the process of tumor budding (Karamitopoulou et al., 2011). This is further underlined by expression of laminin-5γ2 which is supposed to lead to activation of SLUG and ZEB1 (Schmalhofer et al., 2009).

**FIGURE 1:**
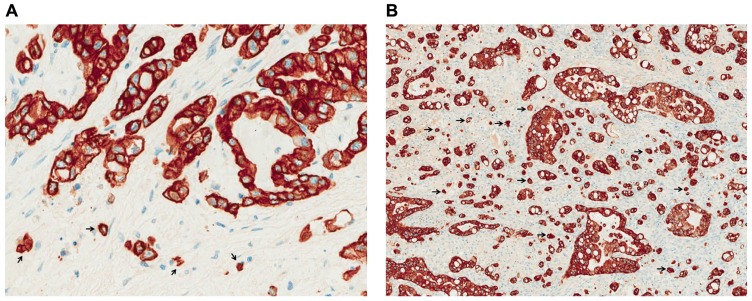
**Low- (A) and high-grade (B) tumor budding in PDAC (pancytokeratin staining, ×400)**. Arrows indicate examples of tumor budding.

In a recent study by our own group the presence and prognostic significance of tumor budding in PDAC were investigated (Karamitopoulou et al., 2012). We found an association between high-grade budding and aggressive clinicopathological features of the tumors, like advanced pT-stage and the presence of lymphatic invasion. Furthermore, we could show that tumor budding occurs frequently in pancreatic cancer and is a strong and independent prognostic factor that can be used as an indicator of patient outcome having a more powerful prognostic ability than other more classic prognostic factors including TNM (Tumor, Node, Metastasis) stage. In more detail, high-grade tumor budding was strongly associated with less overall and disease-free survival, while patients with low-grade budding survived longer and had longer disease-free intervals independently of the presence of other adverse prognostic factors like lymphatic invasion, presence of lymph node metastasis or positive resection margins (**Figures 1A,B**).

## CSCs, EMT-CELLS, BUDDING-CELLS, AND CELL PROLIFERATION

Although cancer cells are often considered as highly proliferative, there is less proliferation at the invasion front of carcinomas (Jung et al., 2001; Carmeliet et al., 2009). Moreover, cells undergoing EMT, just as cells during embryonic development, stop dividing when migrating. A likely explanation is that the cytoskeletal changes occurring during EMT are incompatible with cell division (Barrallo-Gimeno and Nieto, 2005; Richardson et al., 2006; Carmeliet et al., 2009; Giampieri et al., 2009). In support of this, Ki67 labeling was found to be decreased at the invasion front of tumors (Friedl and Gilmour, 2009). Moreover, a transcription factor, inducing EMT and SNAIL was also shown to induce cell cycle inhibitor p21, repress cell cycle activator cyclin D and induce resistance to apoptosis (Kajita et al., 2004; Vega et al., 2004; Perez-Losada et al., 2005). In a breast cancer cell line down-regulation of cyclin A1 was shown to increase migration and decrease proliferation (Lehn et al., 2010).

The relation of CSC- and EMT-properties with cell proliferation is not obvious. Indeed, typical EMT-cells do not proliferate. If CSCs represent dormant cells that proliferate slowly, thus escaping chemotherapy, this is compatible with cells in EMT state (Sell, 2006). If on the other hand, CSCs represent the most aggressive, highly proliferating neoplastic cells, this could be incompatible with EMT state (Yeung et al., 2010). However, the overlap of CSC- and EMT-properties with proliferative activity has not necessarily to be simultaneous. If we assume that the EMT state represents a transient phase in the lifetime of a neoplastic cell, it is likely that the most competitive tumor cells when detached from the others (i.e., tumor budding cells) adopt transiently an EMT state that allows them to invade and metastasize and then, when in their new site, they recover their previous highly aggressive and proliferating nature. In this case, some biomarkers of CSCs or EMT-cells would be expressed only at certain stages of this process (Floor et al., 2011).

Interestingly, in keeping with the previous assumption, in a recent work by our group performed in colorectal cancer, tumor budding cells were shown to have reduced proliferative activity as measured by Ki67, compared with the main tumor (Lugli et al., 2012b).

## CONCLUSION

**FIGURE 2:**
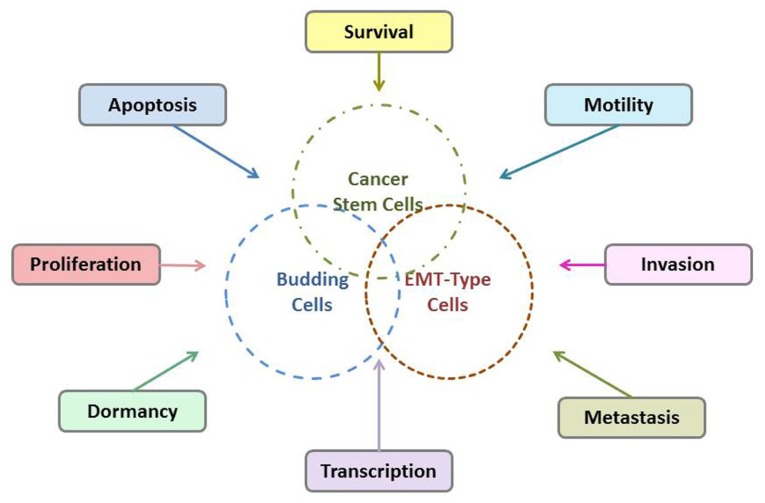
**Possible interactions of tumor budding cells, EMT-type cells, and CSCs**.

Tumor budding is thought to reflect the process of EMT which allows neoplastic epithelial cells to acquire a mesenchymal phenotype thus increasing their capacity for migration and invasion and help them become resistant to apoptotic signals (Guarino et al., 2007; Katoh, 2011). Additionally, it has been suggested that tumor budding cell may have a “stem cell” character. Possible interactions of tumor budding cells, EMT-type cells, and CSCs are shown in **Figure 2**. The WNT pathway which is involved in the process of tumor budding has a strong association with CSCs and the development of a stem cell-like phenotype (Katoh, 2011). Moreover, emerging evidence has shown that CSCs share similar molecular characteristics with EMT-type cells, are drug resistant and have higher metastatic potential (Mani et al., 2008; Morel et al., 2008). In an excellent recent work by Floor et al. (2011) similarities between CSCs and EMT-cells were further explored. It was shown that cancer cells in EMT, that is, EMT-cells, share many properties with the classical so-called “CSC”s. In fact, there are many indications that CSCs present characteristics of EMT-cells and conversely that EMT-cells acquire properties of CSCs, including expression of the markers CD44/CD24, dormancy etc., and vice-versa. The overlap of CSC- and EMT-properties has been also extensively discussed in many recent publications (Alexander et al., 2008; Tomaskovic-Crook et al., 2009; Singh and Settleman, 2010).

However, there is still controversy regarding the relationship between tumor budding cells, EMT-type cells, and CSCs. Characterization studies of the tumor budding cells are very few and so far restricted to immunohistochemical findings. To date it has not been attempted to characterize tumor budding cell at the molecular level. In a previous immunohistochemical study on colorectal cancer from the several potential CSC markers (ABCG5, ALDH1, CD24, CD44, CD90, CD133, EpCam) that have been proposed for solid tumors, only ABCG5 expression in tumor budding cell was found to be associated with poor survival of the patients (Ellenrieder et al., 2001; Visvader and Lindeman, 2008). Further characterization of the tumor budding cells in PDAC on a protein and gene level, especially concerning genes and gene products of the TGF-β and WNT signaling pathways which are promoting EMT- and CSC-features, as well as more detailed exploration of the possible phenotypical and molecular similarities between budding cells, EMT-type cells, and CSCs are needed. Creating a molecular “tumor budding promoting profile” would help to better stratify PDAC patients into prognostic subgroups and to develop possible targets for an individualized therapy.

## Conflict of Interest Statement

The author declares that the research was conducted in the absence of any commercial or financial relationships that could be construed as a potential conflict of interest.
